# The Physiology of Death from Traumatic Fever

**Published:** 1894-09

**Authors:** 


*The Physiology of Death from Traumatic Fever.* By JOHN D. MALCOLM, M.B. Pp. 129. London: J. & A. CHURCHILL. 1893.

The theories advanced in this book are very carefully reasoned out, but the facts supporting them seem to us insufficient.

In general contraction of the arteries Dr. Malcolm sees the explanation of many of the phenomena of traumatic fever. He says: "The pathological interference with the supply of blood to the injured parts causes a reflex physiological contraction of the arteries throughout the rest of the body, and consequently a determination of blood to the inflamed area, which is permitted by local dilatation of the vessels surrounding the damaged parts." The interference, he explains, is due to stasis and exudation in the injured part, the result of partial devitalisation of the tissue. Now the area of stasis and exudation around an operation wound cannot be large. Dr. Malcolm sees this objection himself, and suggests increased irritability of the vaso-motor nerves of the part as an explanation, but we do not consider it a sufficient one.

The production of the high temperature is considered to be due to a reflex stimulation of heat production, by the need of more heat in the tissues "devitalised" by the wound. But no facts are brought forward which support such a theory. We had come to believe that traumatic fever—with the exception perhaps of a few instances such as the slight rise of temperature after simple fracture sometimes observed—was a condition of septic poisoning: sapræmia, in fact, in a mild and transient form. If Dr. Malcolm's theory is right, how is it, we ask, that some of our largest wounds, such as are made by excision of the breast and entire removal of all the fatty and gland contents of the axilla, heal with no rise in temperature whatever if we keep them aseptic?

The most interesting part of this work seems to us to be the section on paralysis of the bowel after abdominal operations.

The book suffers from the absence of an index.

